# Gastrocnemius Medial Head Stiffness Is Associated with Potential Fall Risk in Community-Dwelling Older Adults

**DOI:** 10.3390/healthcare10050785

**Published:** 2022-04-23

**Authors:** Naryeong Kim, Joohwan Park, Heejin Shin, Youngsook Bae

**Affiliations:** Department of Physical Therapy, College of Health Science, Gachon University, 191 Hambangmoe-ro, Yeonsu-gu, Incheon 21936, Korea; kimnr0191@naver.com (N.K.); haking123456@gmail.com (J.P.); shj532351@gmail.com (H.S.)

**Keywords:** balance ability, elderly, fall risk, gastrocnemius muscle, shear wave elastography

## Abstract

The aim of this study is to compare the muscle strength, balance ability, thickness, and stiffness of the tibialis anterior and gastrocnemius muscle in the elderly, with (fallers) and without (non-fallers) fall experience, and confirmed the correlation between the variables mentioned above and muscle stiffness in the faller. We selected 122 elderly participants, comprising 40 fallers and 82 non-fallers, and measured the muscle strength of the tibialis anterior (TA) and the gastrocnemius (GA). Balance ability was measured by the functional reach test (FRT), timed up and go test (TUG), short physical performance battery (SPPB), and gait speed (GS). We used shear wave elastography (SWE) to determine the thickness of the TA and the medial (GAmed) and lateral head (GAlat) of the gastrocnemius and the stiffness during relaxation and contraction. Balance ability, except muscle strength, was significantly lower in fallers compared with non-fallers. The GAmed and GAlat thickness were significantly lower in fallers than that in non-fallers. In fallers, the thickness, rest, and contractive stiffness of GAmed were correlated with the FRT, GS, SPPB. Low rest and GAmed contractive stiffness were related to lower balance ability in fallers. The muscle stiffness measurement using SWE was a novel method to assess potential fall risk.

## 1. Introduction

In older adults, the risk of falling affects their physical ability to maintain independence [1]. These falls often result in serious injuries such as fractures, cerebral hemorrhage etc. These injuries increase the number of unnecessary or preventable hospital stays and put the patients at risk of nosocomial complications. On a wider scale these potentially preventable injuries also place an added burden on the health care system [2,3]. Fall experience reduces physical activity and the quality of life [4]. Falls are caused by loss of balance in static or dynamic postural conditions, such as walking. Aging-related alterations in the visual, vestibular and somatosensory system are known to progressively affect the ability of the elderly to balance [5]. Sarcopenia (a decrease in muscle mass and strength), in particular, is an independent risk factor for falls [6] and a decrease in muscle strength and power, with aging, correlates with poor balance [7]. Early screening can prevent falls and their on sequences in community-dwelling older adults [8]. Particularly, an assessment of muscle performance is essential for the early recognition of physical function decline [9]. Muscle function depends on muscle mass, strength, stiffness, and its contractile properties [9], predominantly in the lower extremities [10]. In addition, the elderly who suffer a fall have, overall, poorer physical function than non-fallers [11]. Therefore, the risk of falls in older adults may be predicted by assessing their balance ability and the muscle strength of their lower extremities [12].

According to a recent study, the stiffness or elasticity of the lower extremity muscles during contraction is correlated with physical functions such as fast walking speed. [13,14]. Using ultrasound, GA thickness has been shown to be associated with low skeletal muscle mass in older adults. [15]. Ultrasound shear wave elastography (SWE) is a novel, quantitative, and non-invasive method to assess muscle stiffness. It may be useful for determining muscle stiffness of contraction intensity [16]. Saito et al. [13] reported that the gastrocnemius muscle (GA) elasticity is correlated to the timed up and go test (TUG) score and decreased maximum walking speed. Balance is controlled by adjustments to the sagittal and frontal plane positions of the foot. In the frontal plane, the tibialis anterior (TA) and peroneus are activated, and in the sagittal plane, the GA and TA are activated [17]. In the elderly, altered TA strength may limit the ability to invoke the lateral ankle in the frontal plane [18] as the TA is crucial for walking and maintaining dynamic balance [19]. However, the peroneus mainly contributes to the lateral ankle’s compensation mechanisms for outwardly directed perturbations. No kinematic differences in peroneus function between the young and the elderly, were found [20]. Therefore, we focused on the TA and the medial (GAmed) and lateral head (GAlat) of the gastrocnemius muscle in older adults. These muscles are responsible for dorsiflexion and plantar flexion of the ankle joint.

Evaluating changes in muscle properties, such as muscle stiffness of the lower extremities rather than muscle strength or physical function, may be more effective in the early recognition of deterioration in balance function in the elderly, such as the risk of fall. Therefore, we aimed to compare the balance ability, muscle strength, thickness and stiffness of the TA, GAmed, and GAlat in community-dwelling and independently living older adults, with (fallers) and without fall experience (non-fallers). In addition, we intended to determine the correlation between the above-mentioned parameters and muscle stiffness in fallers. We hypothesized that the stiffness of the TA, GAmed, and GAlat was lower in fallers than that in non-fallers, considering their decreased physical function and that these differences were associated with a decline in balance ability.

## 2. Materials and Methods

### 2.1. Study Design and Ethical Considerations

We performed a cross-sectional trial. All participants were provided detailed information on the study procedure and safety, and they provided their written informed consent. All study procedures were approved by the Institutional Review Board (1044396-202105-HR-097-01), and the study was conducted in accordance with the tenets of the Declaration of Helsinki. Data were collected from July 2021 to August 2021.

### 2.2. Participants and Procedures

We enrolled 122 older adults (age range, 65–92 years) through various means of community center advertisement, such as posters. The participants were selected by telephonic interviews according to the eligibility criteria. The inclusion criteria were: (1) the ability to independently perform activities of daily living; (2) no history of cardiovascular disease (except hypertension); (3) no history of surgery for musculoskeletal disorders of the lower extremities; and (4) no history of neurological disorders, such as cerebral infarction. (1) Individuals who had a mini-mental state examination score of less than 24; (2) were unable to walk without assistive devices; (3) had premorbid or current orthopedic problems involving the lower extremities; and (4) did not undergo the measurement procedures were excluded.

The participants were contacted one day prior to the study date. On the study day, they completed questionnaires on demographic characteristics and fall experience, including the number of falls in the past year. Then, other than assessing their balance and muscle strength, we performed the mini-mental state examination to screen for cognitive dysfunction. Finally, we measured the muscle thickness and stiffness. All data collection was performed at the university laboratory.

This study used the G*Power 3.1.7 software (University of Kiel, Kiel, Germany) to calculate the sample size, which was determined based on the one-tailed test, power = 0.8, α = 0.05, and effect size = 0.5. The calculated sample size was 102, and a dropout rate of about 20% was considered. A total of 137 elderly participants were recruited; of these, 9 were excluded and 8 did not complete the procedure. The remaining 122 participants were then divided into the experienced (fallers) group and the non-experienced (non-fallers) group.

### 2.3. Outcome Measurement

#### 2.3.1. Mini-Mental State Examination

The cognitive function of participants was evaluated using the Korean version of the Mini Mental State Examination (K-MMSE) [21]. MMSE is a widely used test of cognitive function for the elderly. It includes tests for orientation, attention, memory, language, and visual-spatial skills. For temporal orientation the participants were asked to confirm the year and time in Korea, and the words “plane”, “pencil” and “pine tree” were used in memory evaluation. Each participant’s attention was assessed by sequentially subtracting 7 from 100. The total K-MMSE score was calculated by summing the correct answers for all K-MMSE sub-sections [22]. Test scores range from 0 to 30, scores of 24 or higher indicate no cognitive impairment [23]. The main cognitive functions that contribute to balance control are memory, attention, and orientation [24]. Participants with a K-MMSE score of 24 or higher were selected because elderly persons with cognitive decline have inherently poorer balance, a greater risk of fall and a greater fear of falling [25].

#### 2.3.2. Balance Ability

Balance ability was measured by the functional reach test (FRT), TUG time, short physical performance battery (SPPB) score, and gait speed was use for assessing the fall risk. The FRT represents the maximal distance a person can reach forward beyond the arm’s length while maintaining a fixed base of support in the standing position [26], and it reportedly has predictive validity for the occurrence of falls in older adults [27]. TUG is a simple outcome measure used to examine functional mobility in adults. Moreover, it has excellent intra-rater reliability to determine the risk of falls in the community-dwelling elderly [28]. The SPPB consists of three tests: standing balance, 4 m walk at a typical pace, and five times chair sit-to-stand test (5TSTS). The standing balance tests included tandem, semi-tandem, and side-by-side standing, and each component was considered complete when the participants stands for 10 s. In semi-tandem and side-by-side standing, 1 point was given for standing for more than 10 s and 0 points were given for not being able to stand for 10 s or if not attempted. In tandem stand, 2 points were given for standing for more than 10 s, 1 point for standing for 3 to 9.99 s, and 0 point for standing for less than 3 s or if not attempted. The participants were requested to walk 4 m at their regular pace to assess the 4 m walk. For the five times chair sit-to-stand test, we performed a pre-test; the participants were requested to fold their arms across their chest and stand up from the chair without using the armrest. We measured the time in seconds from the first sitting position to the last standing position on the fifth stand. Each of the three subtests of the SPPB (standing balance, 4 m walk, and 5TSTS) was scored on a scale of 0 to 4, and the individual scores were added for a total score from 0 to 12, with higher scores indicating better function [29]. The SPPB is a tool for the assessment of fall risk in the elderly [30]. The gait speed was measured with a 10-m walking test, commonly used to evaluate the walking speed [31]. The participants were required to walk a distance of 14 m at a self-preferred speed. The first and last 2-m distances were set as the acceleration and deceleration points, respectively, and not included in the evaluation; thus, the gait speed inside the 10 m middle section was evaluated. We assessed the TUG and gait speed in triplicate and recorded the average measurement.

#### 2.3.3. Muscle Strength

**Figure 1:**
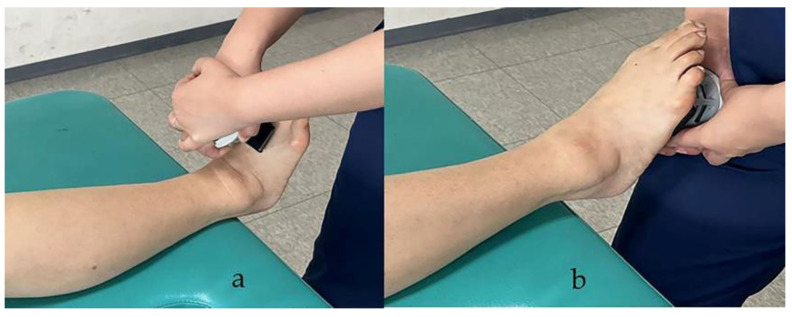
Measurement of muscle strength in tibialis anterior (**a**) and gastrocnemius (**b**).

Muscle strength was measured by maximal isometric spontaneous contractions in the dominant leg using a MicroFET2 handheld dynamometer (Hoggan Industries, Inc., West Jordan, UT, USA). This battery-operated load cell system has a digital readout of the peak force expressed in Newtons (N). The device can select a high or low threshold for the minimum force required to initiate the test. The device was calibrated by the manufacturer prior to the initiation of the study [32]. The maximum isometric strength was measured in the TA and GA as the ankle dorsiflexor and ankle plantar flexor, respectively. First, the participants were shown the movement to be tested. Then, we requested them to perform the movement to confirm their understanding. The contraction interval was measured thrice with a 30 s interval, and the average values were used. The TA was measured with the participant in a supine position; hip and knee extended and ankles off the edge of the examination table. A dynamometer was placed over the dorsum of the foot, just proximal to the toe (Figure 1a) [33,34]. The GA is usually measured by performing ankle plantar flexion, in a standing position, while stepping on a handheld dynamometer. However, in this study the measurement was made in the supine position, as suggested in a previous study, because the participants became unbalanced and could not complete the measurement. The participants were placed in the same position as for the TA measurement and the GA was measured with a dynamometer placed in the middle of the sole of the foot (Figure 1b) [34,35]. Hand-held dynamometry has good to excellent reliability and validity for most measures of isometric lower limb strength [36]. The muscle strength was normalized by bodyweight (N/kg).

#### 2.3.4. Stiffness and Thickness of the Muscle

**Figure 2:**
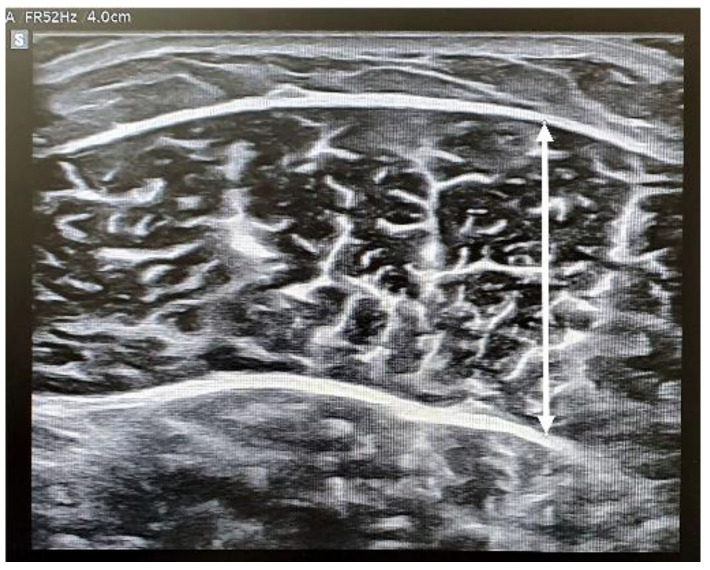
The muscle thickness was measured maximum distance between the fascia.

We measured the thickness, rest, and contraction stiffness of the TA, GAmed, and GAlat of the dominant leg. We measured the thickness of each target muscle in a relaxed state, followed by the rest stiffness. Then, the participants were requested to contract the leg for 10 s to measure the contractive stiffness. Thickness and stiffness were measured using an RS85 ultrasound machine (Samsung Medison, Seoul, Korea) equipped with a 5–10 MHz linear probe. Muscle thickness was measured in cross-section using B-mode (two-dimensional ultrasound image display). Subsequently, we rotated the probe in the direction of the muscle fiber and acquired the rest and contractive muscle stiffness in the longitudinal plane using the SWE mode. Muscle thickness was measured as the distance between the superficial and deep aponeurosis (Figure 2).

**Figure 3:**
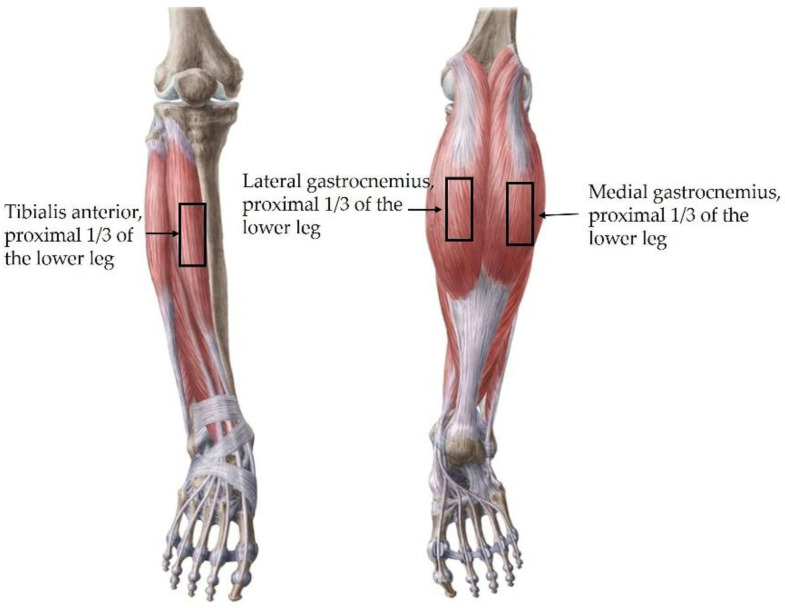
Sites of recording of thickness and stiffness tibials anterior (**left**), and gastrocnemius medial, lateral (**right**).

**Figure 4:**
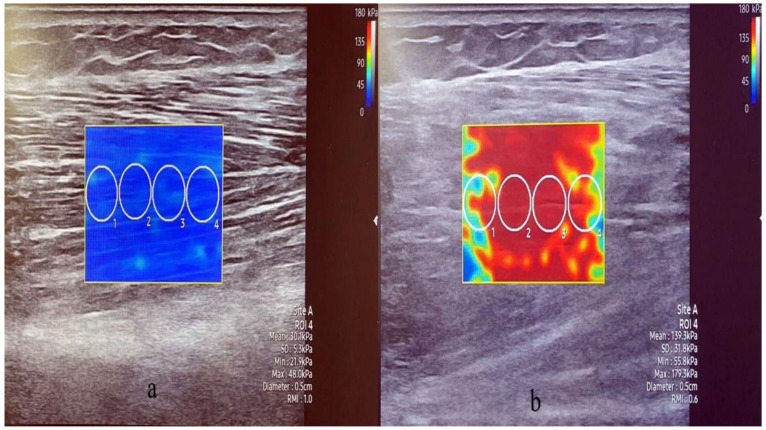
Image of shear wave elastography. The stiffness of medial gastrocnemius was measured under rest state (**a**) and contractive state (**b**).

For muscle stiffness measurement, the participants were allowed to lie comfortably. With the participant in the supine position, the TA muscle stiffness was measured at a point 1/3rd of the distance between the lower margin of the patella and the first metatarsal base (Figure 3 left) [37]. The GA was measured with the participant in the prone position. The Gamed was measured at 1/3rd of the distance from the popliteus medial to the heel, and the GAlat was measured at 1/3rd of the distance from the popliteus, lateral to the heel (Figure 3 right). For the aforementioned muscles, contractive stiffness was measured after rest stiffness measurement and when the participants induced maximum contraction in a similar posture as the muscle strength measurement [14,38]. For muscle stiffness, the circular region of interest was 5 mm in diameter and was placed parallel to muscle fibrils. We avoided focal penetration defects or fibrous septa. Four region-of-interest circles were created in each SWE image, and the average of the stiffness values was calculated (Figure 4) [14]. The values were recorded in Pka. The muscle thickness and stiffness were acquired from the same locations. Ultrasound imaging and SWE were performed concurrently by one of two radiologists with more than 5 years of experience in musculoskeletal radiology.

### 2.4. Statistical Analyses

All statistical analyses were conducted using SPSS version 26 (IBM Corp., Armonk, NY, USA). We analyzed the frequency and used descriptive statistics to assess the participants’ general characteristics. The participants were divided into two groups: fallers (*n* = 40) and non-fallers (*n* = 82). We compared differences in all the variables between the groups using the independent t-test. In fallers, we used Pearson’s correlation test to determine the correlation between physical function and muscle thickness and elastic

## 3. Results

The faller and non-faller groups comprised 40 (mean age: 75.50 years) and 82 (mean age: 73.53 years) participants, respectively. Table 1 summarizes the participants’ characteristics.

### 3.1. Comparison of Physical Function and Muscle Thickness and Stiffness of Fallers and Non-Fallers

**Figure 5:**
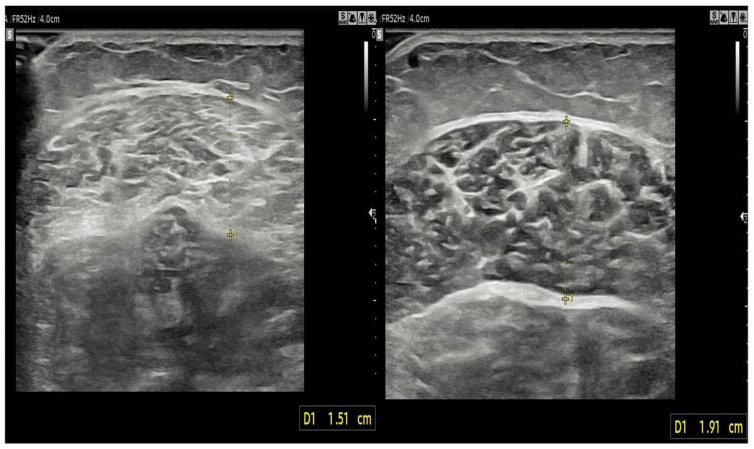
Comparison of thickness of gastrocnemius medialis between faller (**left**) and non-faller (**right**).

**Figure 6:**
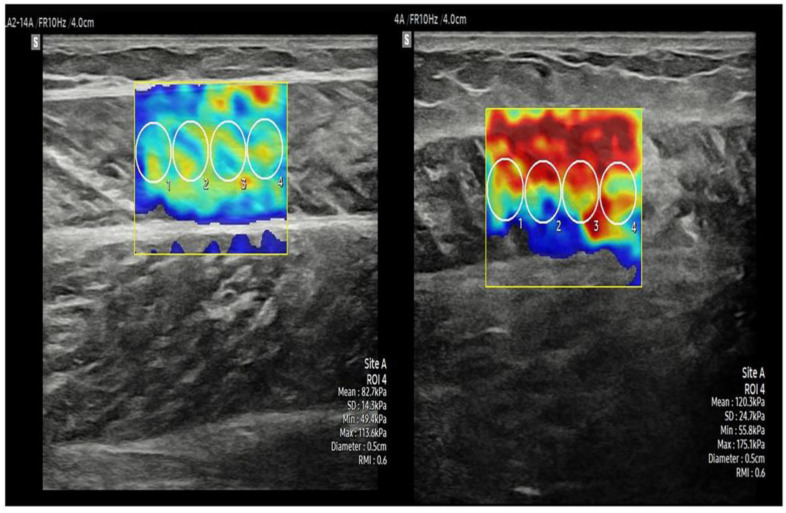
Comparison of contractive stiffness of gastrocnemius medialis between faller (**left**) and non-faller (**right**).

Regarding balance ability, the FRT (*p* = 0.001), TUG (*p* = 0.015), SPPB (*p* = 0.011), and gait speed (*p* = 0.027) were significantly worse in fallers than in non-fallers. There was no difference in the muscle strength between the groups. Regarding muscle thickness and stiffness, the TA rest (*p* = 0.021) and contraction stiffness (*p* = 0.021), GAmed thickness (*p* = 0.008) and contraction stiffness (*p* = 0.002), and GAlat thickness (*p* = 0.014) and contraction stiffness (*p* = 0.006) were significantly lower in fallers compared to non-fallers (Table 2, Figure 5 and Figure 6).

### 3.2. Correlation between Physical Function and Muscle Thickness and Stiffness in Fallers

Table 3 summarizes the correlation between the balance ability and muscle thickness and elasticity of fallers. Gamed thickness was correlated with the FRT (*p* = 0.017, *r* = 0.375) and gait speed (*p* = 0.038, *r* = 0.329). Whereas Gamed rest elasticity was correlated with the SPPB (*p* = 0.034, *r* = 0.337), Gamed contraction elasticity was correlated with the gait speed (*p* = 0.045, *r* = 0.319) and SPPB (*p* = 0.035, *r* = 0.334). However, TA and Galat showed no significant correlations with any of the above parameters.

## 4. Discussion

This study compared differences in the muscle strength, muscle thickness and elasticity of the ankle joint, and balance ability between elderly fallers and non-fallers. Despite no difference in the muscle strength, fallers demonstrated significantly decreased balance ability (FRT, TUG, SPPB, and gait speed), rest, and contractive stiffness of the TA, and thickness and contractive stiffness of the Gamed and Galat. Moreover, their Gamed thickness, rest, and contractive stiffness were correlated with balance ability. Thus, contractive stiffness has a greater association with falls in older adults than muscle strength of lower extremities.

In the elderly, lower extremity strength decreases faster than upper body strength [39]. Decreased muscle strength is associated with physical function limitation [40] and in a recent longitudinal study, maintaining or increasing muscle mass did not prevent muscle strength loss due to aging [41]. Therefore, decreased physical function, which includes falls, was confirmed by the evaluation of lower extremity muscle strength using handheld and fixed dynamometers with good validity and reliability [34]. Falls in older adults are correlated with decreased function of the sensory system, decreased muscle strength in the lower extremities, and reduced cognitive function, balance, and gait ability [42,43].

An FRT ≥ 25.40 cm predicts no risk of fall, compared with a value ranging from 15.24 cm to 25.40 cm that predicts a risk of fall within 6 months [44]. The TUG is another useful tool for assessing impaired balance ability that can increase the risk of falls in the elderly [45], and the risk is higher for TUG scores >13.5 s [46]. The SPPB is significantly associated with fall experience [46], and scores ≤10 may increase the risk of falls [45]. In addition, gait speed <1.0 m/s is significantly associated with a fall history and increased fall risk [47]. In the present study, the FRT mean value was 23.40 cm, TUG was 13.73 sec, the mean SPPB score was 9.57, and gait speed was 1.19 m/s in fallers. In other words, participants in the faller group had reduced balance ability and higher potential fall risk.

Decreasing muscular strength of the lower limb is associated with an increased risk of falling [42]. However, previous studies have reported minimal or no difference in the ankle muscle strength between the faller and non-faller elderly [48]. In addition, muscle stiffness measured using SWE can be used to estimate changes in the muscle force during isometric contractions [49]. Therefore, the risk of falls in the elderly can be more efficiently evaluated by their muscle stiffness rather than muscle strength. Particularly, GA stiffness reflects the change in lower-limb muscle stiffness in the elderly [14]. Our findings suggested that the TA and GA strength were not significantly different between the groups. In contrast, the TA rest and contractive stiffness were significantly lower by 5%, and 6%, respectively, and Gamed, and Galat were significantly lower by 14.2% and 15%, respectively, when comparing fallers to non-fallers. GAmed and GAlat muscle thicknesses were lower by 7% and 10%, respectively, in fallers compared with non-fallers. In addition, GAmed rest and contractive stiffness were related to the gait speed and SPPB scores in fallers. Therefore, GAmed stiffness assessment can be an effective way to identify the risk of falls in the elderly. Particularly, GAmed thickness had a moderate correlation with the FRT and gait speed in fallers. Our results also show that, GAlat thickness and contractive stiffness measurements were lower in the fall group, but there was no correlation with balance ability. The GAmed plays an important role as the ankle plantar flexor during standing balance [50], GAmed activation is greater and longer in duration than GAlat activation during gait [51]. Specifically, GAmed activities and contractile velocity linearly increases as walking speed increases [52,53], and in late in the stance phase of walking, a large force should be induced to accelerate the foot to the next step [53]. Therefore, the decreased GAmed stiffness may be slow the walking speed. Therefore, the author predicts that lower GAmed stiffness than GAlat will have a correlation with balance ability. Ultrasonography-measured GA thickness is associated with low skeletal muscle mass in older adults [15]. Therefore, we identified GAmed stiffness and thickness as predictive factors for balancing ability in older adults.

Therefore, we identified GAmed stiffness and thickness as predictive factors for balancing ability in older adults. Accordingly, an evaluation of muscle elasticity of lower extremities could lead to greater reliability of faller detection among elder population. In addition, in this study, the author predicts that fallers would have a slower walking speed in activity daily of living compared to the non-faller. Therefore, authors proposed that power walking such as walking at a high speed may prevent fall risk while maintaining or improving GAmed activities and contractile velocity in the elderly.

The present study had some limitations. First, previous studies mentioned that the elasticity and thickness of the rectus femoris were associated with dynamic balance and gait speed [13]. This warrants further measurements of the thickness and elasticity of the rectus femoris. Second, we enrolled relatively healthy and active participants and did not control their regular physical activity. Thus, they may not represent a population at risk for falls. Third, the number of faller elderly was small, thus necessitating studies on larger sample sizes. Despite these limitations, this study had several advantages. This is the first study to investigate the possibility of predicting fall risk using the biomechanical properties of muscles by confirming the relationship between a decrease in balance ability and muscle stiffness in the elderly. Therefore, it provides the basis for further studies on SWE and the biomechanical properties of muscles to predict fall risk.

## 5. Conclusions

The low rest and contractive stiffness of GAmed was correlated to lower balance ability in the faller group. The GAmed muscle stiffness can be used to effective methods for predicting potential fall risk in the elderly due to balance deterioration.

## Figures and Tables

**Table 1:** Demographic characteristics and health condition of the participants.

Variables (Unit)	Faller Group (*n* = 40)	Non-Faller Group (*n* = 82)
Demographic characteristics		
Age (year)	75.50 ± 5.80 ^a^	73.53 ± 5.36
Sex	Male: 7 (17.5) ^b^ Female: 33 (26.9)	Male: 20 (24.4) Female: 62 (75.6)
Weight (cm)	59.08 ± 9.42	62.36 ± 10.82
Height (kg)	152.76 ± 6.54	153.41 ± 17.31
BMI ^c^ (kg/m^2^)	25.23 ± 2.95	25.89 ± 3.49
K-MMSE ^d^ (score)	26.15 ± 2.95	26.30 ± 2.72
Number of falls	1.55 ± 1.06	0
Marital status		
Married	24 (60.0)	53 (64.6)
Divorce	3 (7.5)	3 (3.7)
widowed	13 (32.5)	24 (29.3)
Never married	0 (0)	2 (2.4)
Health condition		
Current health condition		
Good	15 (37.5)	33 (40.2)
average	11(27.5)	25 (30.5)
Bad	14 (35.0)	24 (29.3)
Taking medication		
Hypertension	23 (87.5)	56 (68.3)
Diabetes	8 (20.0)	18 (22.0)
Musculoskeletal Disease	6 (15.0)	7 (8.5)
Digestive system disease	2 (2.4)	4 (4.9)
Pain killer	1 (2.5)	2 (2.4)
No	6 (15.0)	12 (14.6)

^a^ mean ± standard deviation, ^b^ number of person (%), ^c^ body mass index, ^d^ Korean Mini mental state examination.

**Table 2:** Comparison of balance ability, muscle strength, and muscle thickness and stiffness in the fallers and non-fallers.

Variables (Unit)	Faller	Non-Faller	*p*	Difference (95% CI)
Balance ability parameter				
Functional reach test (cm)	23.40 ± 6.58	27.52 ± 5.72	0.001	4.127 (6.427~1.828)
TUG (sec)	13.73 ± 3.51	12.31 ± 2.72	0.015	1.425 (0.277~2.574)
SPPB (score)	9.57 ± 1.94	10.48 ± 1.75	0.011	0.901 (0.207~1.594)
Gait speed (m/s)	1.19 ± 0.73	0.98 ±0.26	0.027	0.821 (0.096~1.547)
Muscle strength (N/kg)				
Tibialis anterior	3.06 ± 0.60	3.11 ± 0.74	0.701	0.051 (−0.215~0.319)
Gastrocnemius	2.79 ± 0.56	2.89 ± 0.61	0.424	0.009 (−0.136~0.323)
Muscle thickness and stiffness				
Tibialis anterior				
thickness (mm)	23.72 ± 0.36	24.97 ± 0.33	0.062	0.125 (0.257~0.006)
rest stiffness (kPa)	28.63 ± 6.29	30.37 ± 8.43	0.021	1.745 (−1.235~4.727)
contractive stiffness (kPa)	129.04 ± 22.32	138.15 ± 19.11	0.021	9.113 (16.831~1.395)
Gastrocnemius medial head				
thickness (mm)	17.65 ± 0.27	19.05 ± 0.28	0.008	0.145 (0.037~0.253)
rest stiffness (kPa)	16.29 ± 3.72	16.41 ± 3.66	0.861	0.124 (−1.281~1.530)
contractive stiffness (kPa)	79.72 ± 23.03	92.98 ± 21.01	0.002	13.259 (21.549~4.976)
Gastrocnemius lateral head				
thickness (mm)	14.30 ± 0.29	15.89 ± 0.34	0.014	0.159 (0.284~0.033)
rest stiffness (kPa)	18.14 ± 4.68	17.03 ± 5.66	0.286	1.019 (0.940~3.159)
contractive stiffness (kPa)	90.98 ± 25.25	107.04 ± 31.36	0.006	16.062 (4.790~27.335)

TUG: timed up and go test, SPPB: short physical performance battery.

**Table 3:** Pearson correlations between balance ability muscle thickness and stiffness in the fallers.

Variables	*p* Value	Correlation Coefficient
Gamed thickness vs. FRT	0.017	0.375
Gamed thickness vs. gait speed	0.038	0.329
Gamed rest stiffness vs. SPPB	0.034	0.337
Gamed contraction stiffness vs. gait speed	0.045	0.319
Gamed contraction stiffness vs. SPPB	0.035	0.334

Gamed: Gastrocnemius medial head, TUG: timed up and go test, SPPB: short physical performance battery.

## Data Availability

The data presented in the study are available on request from the corresponding author.
